# Predictors of Pneumothorax in People With COVID-19 Pneumonia: A Multicenter Retrospective Investigation

**DOI:** 10.7759/cureus.38384

**Published:** 2023-05-01

**Authors:** Farooq Dar, Rashid Nadeem, Rawan Albwidani, Fatema Abdulkarim, Mohannad Alassad, Obaid Aljassim

**Affiliations:** 1 Department of Cardiothoracic Surgery, Dubai Health Authority, Dubai, ARE; 2 Intensive Care Medicine, Dubai Hospital, Dubai, ARE; 3 Department of Intensive Care, Dubai Health Authority, Dubai, ARE; 4 Pulmonary Medicine, Rashid Hospital, Dubai, ARE; 5 Department of Cardiothoracic surgery, Dubai Health Authority, Dubai, ARE

**Keywords:** covid-19 pneumonia, intensive care unit (icu), mechanical ventilation, pneumothorax, predictors, uae

## Abstract

This multicenter retrospective investigation aimed to identify predictors of pneumothorax (PTX), pneumomediastinum (PM), and subcutaneous emphysema (SE) in patients with COVID-19 pneumonia admitted to the ICU. A total of 256 patients were included, with 128 in the case group and 128 in the control group. The study sample consisted of predominantly male patients with a mean age of around 53 years and a high prevalence of comorbidities. Significant predictors of PTX, PM, and SE included the presence of coronary artery disease, non-rebreather mask usage, high-flow oxygen therapy, mechanical ventilation, pressor usage, inpatient dialysis, steroid usage, sedative usage, narcotic usage, paralytic usage, elevated C-reactive protein levels, increased lung infiltration, the presence of PM and SE, mode of ventilation, duration of various respiratory support interventions, and severity of illness as indicated by APACHE and SOFA scores. These findings have important implications for the clinical management of patients with COVID-19 pneumonia, as they may help identify and closely monitor at-risk individuals, allowing for timely intervention and potentially improving clinical outcomes. Future research should focus on validating these predictors in larger cohorts and investigating the underlying mechanisms to develop targeted preventive and therapeutic strategies.

## Introduction

The coronavirus disease 2019 (COVID-19), caused by the severe acute respiratory syndrome coronavirus 2 (SARS-CoV-2), has resulted in a global pandemic with a significant impact on healthcare systems worldwide [1]. Pneumonia, a common manifestation of COVID-19, has been associated with a range of complications, including acute respiratory distress syndrome (ARDS), sepsis, and multiorgan dysfunction [2]. Among these complications, pneumothorax (PTX), pneumomediastinum (PM), and subcutaneous emphysema (SE) have emerged as potential complications associated with increased morbidity and mortality in patients with COVID-19 pneumonia [3].

A pneumothorax occurs when air accumulates in the pleural space, leading to lung collapse, while pneumomediastinum is characterized by the presence of air within the mediastinum, and subcutaneous emphysema is defined as air trapped in the subcutaneous tissue [4]. These complications can occur spontaneously or as a result of trauma, lung disease, or mechanical ventilation [5]. In the context of COVID-19 pneumonia, the underlying mechanisms leading to PTX, PM, and SE remain poorly understood, but they may include alveolar damage, barotrauma, and increased intrathoracic pressure secondary to a severe cough [6].

Although several studies have reported the incidence and risk factors of PTX, PM, and SE in patients with COVID-19 pneumonia [7,8], there is still limited information on the predictors of these complications, particularly in the Middle Eastern population. Therefore, this multicenter retrospective investigation aims to identify the predictors of PTX, PM, and SE in patients with COVID-19 pneumonia admitted to the intensive care units (ICU) of Dubai Hospital and Rashid Hospital ICU between January 01, 2020, and December 31st, 2021.

The findings of this study could help improve the clinical management of patients with COVID-19 pneumonia by enabling early identification of those at increased risk of developing PTX, PM, and SE. Furthermore, a better understanding of the predictors of these complications may provide valuable insights into their pathophysiology and facilitate the development of preventive and therapeutic strategies.

## Materials and methods

This study employed a retrospective observational chart review approach, examining all consecutive patients with confirmed COVID-19 who were admitted to the intensive care units (ICU) of Dubai Hospital and Rashid Hospital from January 1, 2020, to December 31, 2021. COVID-19 patients were identified by swabs from the nasopharynx using PCR (polymerase chain reaction) run in our hospital laboratory. Patients who experienced an episode of non-traumatic pneumothorax (PTX), pneumomediastinum (PM), or subcutaneous emphysema (SE) were identified as cases. We used the American Thoracic Society (ATS) published guidelines on the diagnosis of pneumothorax, which clinically defined PTX as the sudden onset of pleuritic chest pain or/and worsening in breathing that may be associated with tachypnea, confirmed by radiological evaluation. Generally, we used chest X-rays with occasional CT scans only if the X-ray findings were equivocal. For pneumomediastinum (PM), we used radiological criteria where a chest X-ray may show a streaky or linear pattern of air within the mediastinum, often most prominent around the heart and great vessels. PM may or may not be associated with clinical changes. Subcutaneous emphysema was clinically diagnosed based on a physical examination revealing bulging of the skin, particularly in areas around the neck, chest, and abdomen, with a characteristic crackling sensation felt when the affected skin was touched due to the presence of air under the skin. Controls were selected from patients with COVID-19 pneumonia who did not develop PTX, PM, or SE, ensuring an equal number of cases and controls. Controls were matched to cases based on age, Sequential Organ Failure Assessment (SOFA) scores, and Acute Physiology and Chronic Health Evaluation-2 (APACHE-2) scores.

Exclusion criteria for this study encompassed pregnant patients, patients with PTX resulting from trauma or as a complication of a procedure, individuals without a COVID-19 infection, pre-existing lung diseases (especially COPD and cystic lung diseases), and coexistent active pulmonary tuberculosis. Potential confounding factors that could influence clinical outcomes were documented, including patient demographics (age, gender, and body mass index [BMI]), comorbidities (diabetes, hypertension, coronary artery disease, renal failure, and outpatient dialysis), and inpatient clinical data at ICU admission (fever, tachycardia, hypotension [systolic blood pressure <90], oxygen [L/min], mechanical ventilation, use of vasopressors, and inpatient dialysis).

Additionally, laboratory parameters such as inflammatory markers (ferritin, C-reactive protein, D-Dimer, and procalcitonin levels) and lactate levels were recorded. Ventilatory parameters and mode of ventilation upon ICU admission (mode of ventilation, level of positive end-expiratory pressure [PEEP], and application of recruitment maneuvers [RM]) were also documented. The severity of illness for each subject was evaluated using SOFA and APACHE-2 scores.

The refined methodology presented here aims to identify predictors of PTX, PM, and SE in patients with COVID-19 pneumonia, providing valuable insights for clinicians to better manage patients at risk for these complications.

## Results

The study sample comprised a predominantly male population with a mean age of around 53 years and a high prevalence of comorbidities such as hypertension and coronary artery disease. There was no significant difference in age or gender distribution between the case and control groups, while the presence of coronary artery disease was significantly higher in the case group. In terms of laboratory findings, C-reactive protein (CRP) levels were significantly higher in the case group, indicating increased inflammation and potentially worse clinical outcomes.

Various modes of ventilation and oxygen support were used in the patients, including non-rebreather (NRB) masks, high-flow nasal cannulas (HFNC), bilevel positive airway pressure (BiPAP), and mechanical ventilation. The use of NRB masks and HFNC was significantly higher in the case group compared to the control group. The proportion of patients receiving mechanical ventilation was also notably higher in the case group compared to the control group. The use of vasopressor agents, which are medications that constrict blood vessels to increase blood pressure, was significantly higher in the case group compared to the control group. This could indicate more severe circulatory issues in the case group.

The proportion of patients requiring inpatient dialysis was significantly higher in the case group compared to the control group, suggesting a higher rate of kidney dysfunction in the case group. The use of corticosteroids, which have been shown to reduce inflammation in severe COVID-19 cases, was significantly higher in the case group compared to the control group. Other medications, such as sedatives and paralytics, were also more frequently used in the case group.

The mean length of stay in the ICU (LOSICU) was significantly longer in the case group compared to the control group, indicating a more prolonged and severe disease course in the case group. The Acute Physiology and Chronic Health Evaluation (APACHE-2) and Sequential Organ Failure Assessment (SOFA) scores are widely used to assess the severity of illness in critically ill patients. The mean APACHE-2 score was significantly higher in the case group compared to the control group, and the mean SOFA score was also significantly higher in the case group compared to the control group. Higher scores in both of these indices indicate a more severe illness in the case group.

In summary, significant predictors of pneumothorax in patients with COVID-19 pneumonia included the presence of coronary artery disease, non-rebreather mask usage, high-flow oxygen therapy, mechanical ventilation, pressor usage, inpatient dialysis, steroid usage, sedative usage, narcotic usage, paralytic usage, elevated C-reactive protein levels, increased lung infiltration, presence of pneumomediastinum and subcutaneous emphysema, mode of ventilation, duration of various respiratory support interventions, severity of illness as indicated by APACHE and SOFA scores, the severity of chest X-ray involvement at presentation, and the rate of deterioration along with increasing respiratory support. These factors should be considered when managing COVID-19 pneumonia patients to reduce the risk of developing pneumothorax and improve clinical outcomes. Table 1 shows data characteristics with p-value.

**Table 1:** Data characteristics along with p-value

Variable	Characteristics	Control (N = 128)	Case ( N=128)	P-Value
Age in years, mean (standard deviation)	53.73 (14.31)	53.50 (13.12)	53.97 (15.46)	0.79
Gender, count (percentage)				0.67
Male	192 (75.00%)	98 (76.56%)	94 (73.44%)	
Female	64 (25.00%)	30 (23.44%)	34 (26.56%)	
Body Mass Index, mean (standard deviation)	30.78 (8.20)	31.12 (8.76)	30.45 (7.61)	0.52
Hypertension, count (percentage)				0.45
No	141 (55.08%)	67 (52.34%)	74 (57.81%)	
Yes	115 (44.92%)	61 (47.66%)	54 (42.19%)	
Coronary Artery Disease, count (percentage)				0.00
No	212 (82.81%)	96 (75.00%)	116 (90.62%)	
Yes	44 (17.19%)	32 (25.00%)	12 (9.38%)	
Prior lung disease, count (percentage)				0.44
No	240 (93.75%)	118 (92.19%)	122 (95.31%)	
Yes	16 (6.25%)	10 (7.81%)	6 (4.69%)	
Chronic Obstructive Pulmonary Disease, count (percentage)				0.11
No	241 (94.14%)	124 (96.88%)	117 (91.41%)	
Yes	15 (5.86%)	4 (3.12%)	11 (8.59%)	
Immune suppression, count (percentage)				0.34
No	224 (87.50%)	109 (85.16%)	115 (89.84%)	
Yes	32 (12.50%)	19 (14.84%)	13 (10.16%)	
Smokers, count (percentage)				0.09
No	243 (94.92%)	125 (97.66%)	118 (92.19%)	
Yes	13 (5.08%)	3 (2.34%)	10 (7.81%)	
Non-Rebreather mask, count (percentage)	4.03 (5.76)	7.24 (6.72)	0.81 (0.82)	0.00
High flow oxygen, mean (standard deviation)	0.73 (0.86)	0.60 (0.49)	0.85 (1.09)	0.02
Bilevel Positive Airway Pressure, count (percentage)				0.51
No	172 (67.19%)	89 (69.53%)	83 (64.84%)	
Yes	84 (32.81%)	39 (30.47%)	45 (35.16%)	
Mechanical ventilation, count (percentage)				0.00
Yes	153 (59.77%)	28 (21.88%)	125 (97.66%)	
No	103 (40.23%)	100 (78.12%)	3 (2.34%)	
Vasopressors, count (percentage)				0.04
Yes	236 (92.19%)	113 (88.28%)	123 (96.09%)	
No	20 (7.81%)	15 (11.72%)	5 (3.91%)	
Dialysis, count (percentage)				0.00
Yes	136 (53.12%)	93 (72.66%)	43 (33.59%)	
No	120 (46.88%)	35 (27.34%)	85 (66.41%)	
Steroids, count (percentage)				0.00
Yes	192 (75.00%)	69 (53.91%)	123 (96.09%)	
No	64 (25.00%)	59 (46.09%)	5 (3.91%)	
Tocilizumab, count (percentage)				0.76
Yes	203 (79.30%)	103 (80.47%)	100 (78.12%)	
No	53 (20.70%)	25 (19.53%)	28 (21.88%)	
Sedatives, count (percentage)				0.00
Yes	175 (68.36%)	50 (39.06%)	125 (97.66%)	
No	81 (31.64%)	78 (60.94%)	3 (2.34%)	
Narcotics, count (percentage)				0.05
Yes	242 (94.53%)	117 (91.41%)	125 (97.66%)	
No	14 (5.47%)	11 (8.59%)	3 (2.34%)	
Paralytics, count (percentage)				0.00
Yes	233 (91.02%)	108 (84.38%)	125 (97.66%)	
No	23 (8.98%)	20 (15.62%)	3 (2.34%)	
Ferritin levels, mean (standard deviation)	7824.43 (34128.90)	7917.87 (44138.96)	7730.99 (19760.31)	0.97
C-Reactive Protein, mean (standard deviation)	770.32 (2252.07)	1263.52 (3092.69)	277.13 (358.58)	0.00
Infiltrates, mean (standard deviation)	50.10 (94.26)	99.20 (113.93)	1.00 (0.00)	0.00
Pneumomediastinum subcutaneous, count (percentage)				0.00
No	141 (55.08%)	48 (37.50%)	93 (72.66%)	
Yes	115 (44.92%)	80 (62.50%)	35 (27.34%)	
Pneumothorax, count (percentage)				0.00
No	148 (57.81%)	128 (100.00%)	20 (15.62%)	
Yes	108 (42.19%)	0(0%)	108 (84.38%)	
Subcutaneous emphysema, count (percentage)				0.00
No	214 (83.59%)	128 (100.00%)	86 (67.19%)	
Yes	42 (16.41%)	0(0%)	42 (32.81%)	
Pneumomediastinum + pneumothorax both, count (percentage)				0.00
No	228 (89.06%)	128 (100.00%)	100 (78.12%)	
Yes	28 (10.94%)	0(0%)	28 (21.88%)	
Day number of pneumomediastinum/pneumothorax, mean (standard deviation)	9.79 (14.93)	0.00 (0.00)	19.58 (15.94)	0.00
Mode of ventilation, count (percentage)				0.00
Synchronized Intermittent Mandatory Ventilation	110 (42.97%)	19 (14.84%)	91 (71.09%)	
Non-Invasive Ventilation	23 (8.98%)	6 (4.69%)	17 (13.28%)	
Pressure Control	22 (8.59%)	18 (14.06%)	4 (3.12%)	
High flow oxygen	14 (5.47%)	4 (3.12%)	10 (7.81%)	
Airway Pressure Release Ventilation	3 (1.17%)	0(0%)	3 (2.34%)	
Adaptive Support Ventilation	2 (0.78%)	0(0%)	2 (1.56%)	
Days on high flow oxygen, mean (standard deviation)	3.27 (5.01)	1.91 (3.33)	4.62 (5.97)	0.00
Number of days without BIPAP, mean (standard deviation)	2.34 (6.28)	1.47 (3.22)	3.20 (8.21)	0.03
Days on mechanical ventilation, mean (standard deviation)	16.99 (27.87)	6.96 (17.12)	27.02 (32.62)	0.00
Length of Stay in Intensive Care Unit, mean (standard deviation)	24.91 (27.25)	18.92 (15.30)	30.89 (34.43)	0.00
Acute Physiology and Chronic Health Evaluation score, mean (standard deviation)	18.80 (13.96)	10.76 (15.03)	26.85 (5.92)	0.00
Sequential Organ Failure Assessment score, mean (standard deviation)	13.06 (5.51)	15.61 (6.46)	10.51 (2.50)	0.00

## Discussion

The results of this multicenter retrospective investigation provide valuable insights into the predictors of PTX, PM, and SE in patients with COVID-19 pneumonia admitted to the ICU. The findings of this study can be utilized to improve the clinical management of these patients by identifying those at an increased risk of developing these complications, thus potentially reducing morbidity and mortality associated with COVID-19 pneumonia.

Our findings show that patients with COVID-19 pneumonia who developed PTX, PM, and SE were more likely to have coronary artery disease, require a higher level of respiratory support, and experience more severe disease, as evidenced by higher APACHE-2 and SOFA scores. These results are consistent with previous studies that have identified similar risk factors for the development of PTX, PM, and SE in patients with COVID-19 pneumonia [9,10]. The increased use of NRB masks, HFNC, and mechanical ventilation in the case group suggests that more severe respiratory failure and the need for higher levels of respiratory support may predispose patients to PTX, PM, and SE. This is in line with the notion that barotrauma from mechanical ventilation and increased intrathoracic pressure due to severe coughing may contribute to the development of these complications [6].

The elevated CRP levels observed in the case group indicate that inflammation may play a role in the development of PTX, PM, and SE in patients with COVID-19 pneumonia. Previous research has shown that increased inflammation, as indicated by elevated CRP levels, is associated with worse clinical outcomes in COVID-19 patients [11]. The exact mechanism by which inflammation contributes to the development of PTX, PM, and SE remains unclear and warrants further investigation.

The presence of coronary artery disease as a significant predictor of PTX, PM, and SE may be related to the known association between COVID-19 and cardiovascular complications, including myocardial injury, thrombosis, and acute coronary syndrome [12]. Additionally, the systemic inflammation induced by SARS-CoV-2 infection may exacerbate pre-existing cardiovascular conditions, increasing the risk of developing PTX, PM, and SE [13].

The use of vasopressors, corticosteroids, sedatives, and paralytics was also more frequent in the case group, suggesting that these patients experienced more severe disease and required additional pharmacological interventions to manage their condition. Furthermore, the longer LOSICU in the case group indicates a more prolonged and severe disease course, which may increase the risk of developing PTX, PM, and SE.

The findings of this study should be interpreted in light of some limitations. First, as a retrospective study, the investigation may be subject to selection bias, and causality cannot be inferred from the observed associations. Second, the sample size is relatively small, which may limit the generalizability of the findings. Further research in larger cohorts is necessary to confirm the predictors identified in this study and explore potential mechanisms underlying their association with PTX, PM, and SE in patients with COVID-19 pneumonia.

## Conclusions

This multicenter retrospective study identified key predictors of pneumothorax, pneumomediastinum, and subcutaneous emphysema in ICU-admitted COVID-19 pneumonia patients, including coronary artery disease, respiratory support interventions, elevated inflammatory markers, and higher severity of illness scores. These findings have crucial implications for clinical management, as they can help healthcare professionals monitor at-risk individuals and enable timely interventions to potentially improve outcomes. Future research should validate these predictors in larger, more diverse patient populations and investigate the mechanisms behind these complications. This knowledge could lead to targeted preventive and therapeutic strategies. Additionally, exploring alternative respiratory support and optimizing mechanical ventilation settings can help minimize complications in severe cases.

Interdisciplinary collaboration is essential for optimal patient management, enabling early recognition and timely intervention for patients at risk. Overall, this study aids clinicians in identifying patients at increased risk, with the potential to improve clinical management and reduce morbidity and mortality. Further research should focus on understanding the underlying associations and developing preventive and therapeutic strategies for at-risk patients.
