# Correction: Predictors of Pneumothorax in People With COVID-19 Pneumonia: A Multicenter Retrospective Investigation

**DOI:** 10.7759/cureus.c237

**Published:** 2025-08-11

**Authors:** Farooq Dar, Rashid Nadeem, Rawan Albwidani, Fatema Abdulkarim, Mohannad Alassad, Obaid Aljassim

**Affiliations:** 1 Department of Cardiothoracic Surgery, Dubai Health Authority, Dubai, ARE; 2 Intensive Care Medicine, Dubai Hospital, Dubai, ARE; 3 Department of Intensive Care, Dubai Health Authority, Dubai, ARE; 4 Pulmonary Medicine, Rashid Hospital, Dubai, ARE; 5 Department of Cardiothoracic surgery, Dubai Health Authority, Dubai, ARE

The Ethics Statement and Conflict of Interest Disclosures section of this article has been corrected to indicate that this study did involve human subjects or tissue.

